# Supplementary material for: Phase-separated droplets swim to their dissolution
Source: Nat Commun. 2024 May 9;15:3919. doi: 10.1038/s41467-024-47889-y (PMC11082165; doi:10.1038/s41467-024-47889-y)
Supplement: Supplementary file 2 — Description of Additional Supplementary Files [file 41467_2024_47889_MOESM2_ESM.pdf]

Title: Supplementary Movie 1:
Description: Definition of the PEG-BSA phase separated system and experimental movies of active PEG-BSA droplets swimming towards each other on a PEGDA 12k coating.

Title: Supplementary Movie 2:
Description: Schematic of the gradient chamber and movies showing internal flows of passive PEG-BSA droplets in various external chemical gradients.

Title: Supplementary Movie 3:
Description: Movie of an active PEG-BSA droplet slowly dissolving.

Title: Supplementary Movie 4:
Description: Movies of passive PEG-BSA droplet swimming and dissolving in extreme external gradients.
